# Assessment of intraoperative soft tissue balance in functional alignment total knee arthroplasty: A comparative study with anatomic alignment

**DOI:** 10.1002/jeo2.70315

**Published:** 2025-07-02

**Authors:** Seikai Toyooka, Hironari Masuda, Noriaki Arai, Yutoshi Osaki, Shuji Ando, Hirotaka Kawano, Takumi Nakagawa

**Affiliations:** ^1^ Department of Orthopaedic Surgery Teikyo University School of Medicine Tokyo Japan; ^2^ Department of Information Engineering Tokyo University of Science Tokyo Japan

**Keywords:** anatomical alignment, functional alignment, mechanical alignment, robotic‐assisted surgery, total knee arthroplasty

## Abstract

**Purpose:**

The purpose of this study is to evaluate the gap balance after implantation in patients who have undergone total knee arthroplasty (TKA) via functional alignment (FA) by comparing the virtual gap in preoperative simulation. To facilitate the evaluation of balance characteristics, a control group consisted of patients who underwent anatomical alignment (AA) with the articular surfaces tilted medially and set up in neutral alignment.

**Methods:**

We retrospectively analyzed data from 321 consecutive knees of patients with varus knee osteoarthritis who underwent primary cruciate‐retaining TKA from September 2017 to July 2023. The FA group included 140 knees that were operated on via a robotic arm based on kinematic alignment, and the AA group included 93 knees that were operated on via a navigation system. The two groups were stratified according to the timing of surgery that corresponded to changes in the surgeon's surgical technique. After osteotomy, the implant gap was measured via a digital tensioner at 0°, 30°, 60°, 90° and 120° flexion angles with 30 pounds of tension stress applied, and the gaps were compared between the two groups.

**Results:**

The gap between the implants in the medial compartment was stable in FA, with no widening of more than 2 mm, including the mid‐flexion position. In AA, the gap tended to open with flexion and was significantly larger than in FA at 60° (FA: 1.1 ± 1.5 mm, AA: 1.8 ± 1.9 mm), 90° (FA: 1.2 ± 1.5 mm, AA: 2.2 ± 2.0 mm) and 120° (FA: 1.4 ± 1.6 mm, AA: 2.8 ± 2.1 mm) (*p* < 0.05). In the lateral compartment, the gap was significantly larger in AA than in FA at all the angles (*p* < 0.05).

**Conclusion:**

Compared with AA, FA achieved balance at all knee flexion angles. FA can achieve a well‐balanced knee with high reproducibility and may allow the surgeon to consistently obtain better balance.

**Level of Evidence:**

Level III prospective cohort study.

**Abbreviations**
AA‐TKA: anatomic alignment total knee arthroplasty
FA: functional alignment
KA: kinematic alignment
LDFA: lateral distal femoral angle
MA: mechanical alignment
MPTA: medial proximal tibial angle
SEA: surgical epicondylar axis
TKA: total knee arthroplasty

## INTRODUCTION

In total knee arthroplasty (TKA), there are several methods known to restore alignment and balance, both of which are important factors to ensure an optimal outcome [19, 21]. The current gold standard alignment technique is the use of mechanical alignment (MA), which is thought to have the advantage of providing more durability because the load is applied perpendicular to the loading axis [9]. Recent studies [13, 14, 28] have reported unsatisfactory results in 20% of TKA patients, and one of the reasons for this is thought to be that not all knees are placed in neutral alignment.

In contrast, the idea of recreating prearthritic bony anatomy of the knee has become popular in recent years. This technique, advocated by Howell and referred to as kinematic alignment (KA), is expected to result in more natural motion, better soft tissue balance, and increased patient satisfaction [1, 11]. However, the constitutional and morphological characteristics of the knee are difficult to reliably predict and reproduce, and at the same time, the soft tissue balance changes with osteoarthritis progression [20]. Therefore, even if this alignment technique is performed perfectly, it is difficult to achieve good soft tissue balance without soft tissue procedures in all cases [5].

In this context, functional alignment (FA) has recently been described in the literature as an alternative alignment method [2, 5]. FA is a new technique that adjusts alignment to achieve better soft tissue balance. This is performed with robotic arms, which have rapidly become popular in recent years. Based on the patient's bone morphology obtained by computed tomography (CT) or other means and intraoperative soft tissue balance, the osteotomy and component placement are simulated prior to the actual osteotomy to achieve optimal balance. Clark et al. [5] and Van de Graaf et al. [26] simulated the balance between the extension and flexion positions before the actual osteotomy in TKA for FA and reported that FA is better balanced than MA or KA. Recently, Erard et al. [7] evaluated the balance after FA at each flexion angle by pressure sensor. However, no studies have reported the actual gap balance after implantation using a tensioner in FA. The purpose of this study is to intraoperatively evaluate the gap balance after implantation at 0°, 30°, 60°, 90° and 120° flexion in patients who have undergone TKA with FA by comparing the virtual gap in the pre‐osteotomy simulation. A control group was set up to facilitate the evaluation of balance characteristics. This group consisted of patients who had undergone anatomical alignment (AA), in which the articular surfaces were tilted medially and the alignment was neutral. We hypothesize that FA provides superior soft tissue balance compared to AA, leading to more stable knee mechanics postoperatively.

## MATERIALS AND METHODS

### Patients and design

The study protocol was approved by the institutional review board of the author's institution, and all patients provided informed consent. The control group comprised patients who underwent the anatomic alignment TKA (AA‐TKA) technique, in which osteotomies of the femur were performed at 2° of valgus and the tibia at 2° of varus with neutral overall alignment, and the results were compared with those of the FA group. The two groups were stratified according to the timing of the surgery. Data from 321 consecutive knees in patients with varus knee osteoarthritis who underwent primary cruciate retaining type TKA (Triathlon; Stryker Corporation) from September 2017 to July 2023 at a single institution were analyzed retrospectively. All the surgeries were performed by four orthopaedic staff surgeons under the supervision of one expert surgeon. The FA group included 140 knees that underwent surgery between June 2021 and July 2023 and were operated on with a robotic arm (Mako; Styker Mako) based on kinematic alignment. The AA group included 93 knees that underwent surgery between September 2017 and October 2020 and were operated primarily with a navigation system (Precision version 4.0; Stryker Orthopaedics). The period between the two periods was excluded because surgeries were performed via a different alignment technique. The exclusion criteria included osteonecrosis, PCL dysfunction, severe range of motion limitation (flexion contracture >30°), rheumatoid arthritis, presence of extra‐articular deformity of the femur or tibia, prior osteotomy of the femur or tibia, prior intra‐ or extra‐articular fracture of the femur or tibia, and prior hip replacement surgery. As a result, 88 knees were excluded. All cases that met the criteria were allocated to either of the groups. Implantation gaps measured with a digital tensioner after osteotomy were compared in both groups.

### Surgical procedure

FA was performed via Mako software (Stryker Mako). Preoperative CT scans of the lower extremity were used to define the centre of the hip and ankle joints, along with the creation of a three‐dimensional (3D) model of the knee. The system incorporates knee joint bone geometry and implant information in its software, allowing alignment, amount of resection, and implant size to all be optimally tailored to the surgeon's preferences. The implants were temporarily placed preoperatively in software with restricted kinematic alignment. The plan was made under restricted conditions and bordered by 4° of varus to 2° of valgus in the tibia and 4° of valgus to 2° of varus in the femur in coronal alignment based on the original CT image [1]. As a result, lower extremity alignment in the coronal plane was planned with limitations to within 4° of varus or valgus. The osteotomy angles in the coronal plane of the tibia and femur were determined via a previously reported method [18, 24]. The medial proximal tibia angle (MPTA) between the tibial mechanical axis and the knee joint line of the tibia, and the lateral distal femoral angle (LDFA) between the femoral mechanical axis and the knee joint line of the femur were obtained from preoperative CT, and those cases exceeding the limits were controlled within the limits. The osteotomy volume was planned in terms of the thickness of the implant (femur: 8.5 mm, tibia: 9 mm) from the most distal medial or lateral femoral condyle. Femoral rotation was set parallel to the posterior condyle axis and was adjusted within the range of 0–5° of internal rotation with respect to the surgical epicondylar axis (SEA), which is a line connecting the sulcus of the medial epicondyle and the lateral epicondylar prominence. Femoral flexion was adjusted between 0 and 6 to optimize the femoral component size. The amount of tibial osteotomy was also set to be the thickness of the implant from the most proximal. The tibial posterior slope was set between 0 and 10 based on the original tibial lateral plateau, and rotation was set by the Akagi line.

Penetrating periosteal pins were implanted in the tibia and femur, and an array was attached for Mako. A medial parapatellar approach was used in all patients, with minimal medial soft tissue dissection, and the ACL was resected. Bone landmarks were registered and validated against the patient's 3D CT model by planning software (Stryker Mako). The osteophytes were then removed. At this point, a soft tissue balancing procedure called the FA technique was performed. Stress was applied to the knee medially and laterally in extension (10° to tension the posterior capsule) and medially and laterally in flexion (90°). Manual stress was applied to cause the medial or lateral gap to open as much as possible. The software was used to measure the virtual gap between both the medial and lateral virtual implants. Based on these gap values, the component positioning was adjusted on the monitor screen. The objective was to achieve a balance with equal medial and lateral gaps in extension and a medial gap in flexion, with the thinnest possible tibial insert thickness of 9 mm. Because the lateral gap in flexion has been reported to be larger in the normal knee, the opening of the flexion‐lateral gap was disregarded [5, 12]. Specifically, the osteotomy alignment, osteotomy volume, implant size, and femoral flexion angle were adjusted for the distal femur and proximal tibia. Adjustments were made within the limits of the same boundaries as the initial plan, and osteotomies were performed with Mako accordingly. No additional soft tissue release or other manipulations were performed.

In the AA group, Stryker navigation (Precision version 4.0; Stryker Orthopaedics) was used for all knees. Navigation pins were inserted into the femur and tibia, which were exposed similarly to those used in FA, and the osteophytes and ACL were resected. The functional axes of the femur and tibia were identified by capturing the femoral head centre, knee joint centre, and ankle joint centre, respectively. In all cases, a guide was placed according to the registered bony surface information, and osteotomies were performed so that the distal femur was in 2° of valgus and the proximal tibia was in 2° of varus. The femoral and tibial osteotomies were set to the thickness of the implant from the medial and lateral apexes. After the initial osteotomy of the distal femur and proximal tibia, the medial gaps in extension and flexion were measured with a tensioner. From these values, the amount of osteotomy of the posterior femoral condyle was determined by calculating the medial gap in extension to be equal to the medial gap in flexion. Femoral rotation was set parallel to the SEA, and tibial rotation was determined according to the Akagi line. The posterior tibial tilt was determined according to the original tibial slope.

**Figure 1:**
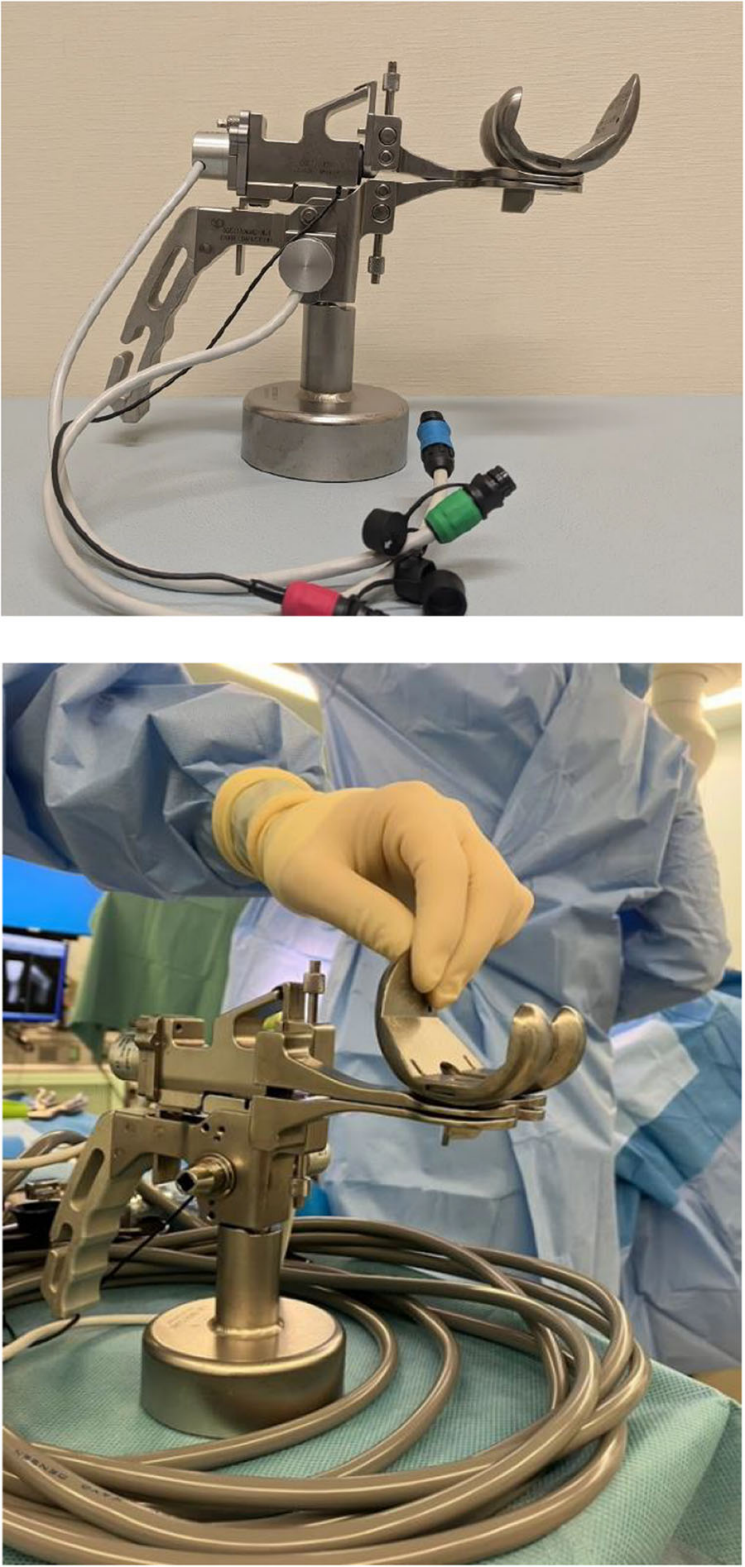
Photograph of the digital tensioner. The tibial side has a keel, and the femoral implant was inserted and measured after the tibial side tensioner was inserted. Gap values for the medial, central and lateral compartments are automatically displayed when a tensile stress of 30 pounds is reached.

Prior to implant insertion, soft tissue balance was assessed in both groups using a digital tensioner (DynAccurate, Stryker Corporation) (Figure 1) [23]. This tensioner is a ‘seesaw’ type and can accurately evaluate the medial, centre and lateral component gap values (mm), and the gap slope is also displayed digitally. After the tibial keel of the digital tensioner was fixed to the tibia implant position, the trial implant was placed on the femoral side. The gap values of the medial, lateral, and central compartments were then evaluated when the joint was opened at 0°, 30°, 60°, 90° and 120° with 30 pounds of tensile stress. Gaps were evaluated for the medial compartment, central position, and lateral compartment. Evaluations were performed with the patella in a reduced position and the knee joint simultaneously maintained in a neutral position with the leg holder.

The measured gap values were compared in both groups for each knee angle. In this study, the target value for the medial gap was 0 mm in both groups. Referring to the study by Clark et al. [5], a balanced knee was defined as a case where the medial compartment gap was 2 mm or less at each knee angle. Although the lateral compartment is known to be more lax than the medial compartment, the ideal value is still unknown; therefore, the lateral compartment was used as a reference value in this study [6]. Box‐and‐whisker plots of the two groups of gap data were created, and the t‐test was used to investigate the differences between the two groups after confirming that the data did not deviate from normal distribution. The statistical significance level was set at *p* = 0.05, and all calculations were performed using SPSS version 12 (SPSS Inc.). To adjust for multiple comparisons, the Bonferroni method was used for reanalysis. Although a power analysis was not conducted prior to this study, a power evaluation was conducted to confirm that the number of cases was sufficient.

## RESULTS

Patient demographics are shown in Table 1. In the FA group, the mean age was 75.9 years (37 knees, men; 103 knees, women). As a result, the femurs were osteotomized with an average of 1.0° of valgus, and the tibias were osteotomized with an average of 3.5° of varus. The average degree of intraoperative alignment of the hip–knee–ankle angle (HKA) was 2.5° of varus. Femoral rotation was osteotomized with an average of 0.9° of internal rotation relative to the SEA. In the AA group, the mean age was 75.3 years (10 knees, men: 83 knees, women). In all cases, the femur was osteotomized with 2° of valgus, and the tibia was osteotomized with 2° of varus, resulting in an intraoperative HKA of 0. Femoral rotation was osteotomized parallel to the SEA in all cases.

**Table 1:** Patient demographics.

	FA‐TKA	AA‐TKA	*P* value	Confidence interval	Effect size (Cohen's *d*)
*n*	140	93			
Sex	Men: 37 knees, Women: 103 knees,	Men: 10 knees, Women: 83 knees,			
Age	75.9 ± 9.2	75.3 ± 8.8	0.57	[−1.59 to 2.88]	0.76
Equipment used	Robotic arm (Mako)	Navigation system			
Preoperative mean LDFA (degree)	88.5 ± 2.0	88.6 ± 2.3	0.84	[−0.58 to 0.47]	0.27
Preoperative mean MPTA (degree)	84.0 ± 2.4	84.0 ± 3.5	0.73	[−0.59 to 0.83]	0.45
Preoperative mean aHKA (degree)	varus 5.0 ± 2.6	Varus 5.1 ± 2.8	0.86	[−0.72 to 0.60]	0.23
Femoral osteotomy angle (degree)	Valgus 1.0 ± 1.4	Valgus 2.0 a	<0.001	[0.71–1.28]	0.92
Tibial osteotomy angle (degree)	Varus 3.5 ± 0.8	Varus 2.0 a	<0.001	[1.10–1.57]	1.52
Intraoperative HKA (degree) b	Varus 2.5 ± 1.5	Neutral a	<0.001	[1.97–2.71]	1.66
Femoral rotation (degree) from SEA	Internal rotation 0.9 ± 1.9	0 a	<0.001	[0.54–1.27]	0.66

Abbreviations: AA‐TKA, anatomic alignment total knee arthroplasty; aHKA, arithmetic hip–knee–ankle angle; FA‐TKA, functional alignment total knee arthroplasty; LDFA, lateral distal femoral angle; MPTA, medial proximal tibial angle; SEA, surgical epicondylar axis.

a As measurements were not taken after the surgery, these are theoretical values.

b Values expressed by the robotic assistance and navigation systems during surgery.

**Figure 2:**
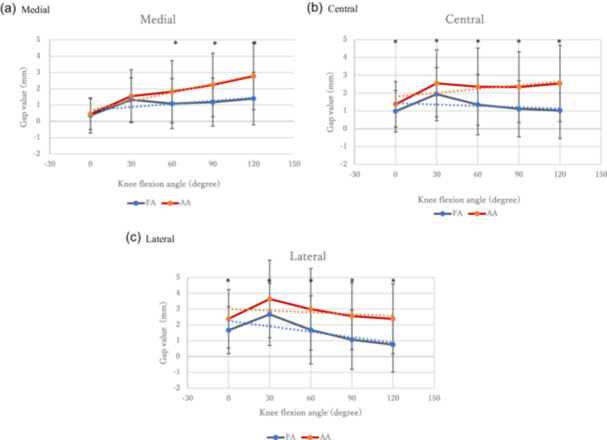
Implant gap value at the knee flexion angle measured via a digital tensioner. (a) Medial compartment, (b) central compartment and (c) lateral compartment. AA, anatomic alignment; FA, functional alignment.

Implant gap values at knee flexion angles measured using a digital tensioner are shown in Figure 2 and Table 2. In terms of the mean implant gap in the medial component, the balance was obtained at all knee flexion angles on FA, with no extreme mid‐flexion gap. In AA, balance was achieved at 0° and 30°; however, the gap tended to open with flexion. There were significant differences between FA and AA at 60°, 90° and 120°. There was also a difference in the proportion of balanced gaps of 2 mm or less at 60°, 90° and 120° (Table 2). In the central gap, the gap of FA opened slightly at 30°, but did not open at other angles. At 60°, 90° and 120°, AA opened significantly more than FA. In the lateral compartment gap, AA was also significantly more open at all angles than FA.

**Table 2:** Details of implant gap data.

(a) Medial					
Knee flexion angle	0	30	60	90	120
Mean implant gap in FA (mm)	0.36 ± 1.08 (−2.3 to 3.1)	1.32 ± 1.35 (−1.3 to 4.8)	1.09 ± 1.54 (−1.5 to 4.9)	1.18 ± 1.47 (−1.1 to 4.2)	1.40 ± 1.61 (−0.9 to 4.5)
Mean implant gap in AA (mm)	0.45 ± 0.95 (−1.2 to 3.3)	1.55 ± 1.63 (−1.2 to 6.0)	1.82 ± 1.91 (−0.8 to 5.8)	2.24 ± 1.95 (−1.0 to 7.9)	2.78 ± 2.06 (0.0–8.7)
*p*	0.534	0.253	<0.001	<0.001	<0.001
Confidence interval (CI)	[−0.19 to 0.37]	[0.17–0.62]	[0.28–1.19]	[0.62–1.52]	[0.89–1.86]
Achievement rate of balance within 2 mm in FA	128/140 (91%)	100/140 (71%)	114/140 (81%)	110/140 (79%)	103/140 (74%)
Achievement rate of balance within 2 mm in AA	88/93 (94%)	65/93 (70%)	57/93 (61%)	46/93 (49%)	42/93 (45%)

(b) Central					
Knee flexion angle	0	30	60	90	120
Mean implant gap in FA (mm)	0.98 ± 1.16 (−1.2 to 4.2)	1.94 ± 1.49 (−1.0 to 5.4)	1.35 ± 1.69 (−1.7 to 5.6)	1.11 ± 1.57 (−1.5 to 5.2)	1.02 ± 1.57 (−1.3 to 5.3)
Mean implant gap in AA (mm)	1.37 ± 1.28 (−0.1 to 3.6)	2.55 ± 1.89 (0.1–6.7)	2.36 ± 2.17 (−3.3 to 6.5)	2.34 ± 1.99 (−0.4 to 5.0)	2.54 ± 2.14 (−0.3 to 5.8)
*p*	0.017	0.008	<0.001	<0.001	<0.001
CI	[0.07–0.72]	[0.16–1.06]	[0.49–1.51]	[0.76–1.70]	[1.03–2.00]

(c) Lateral					
Knee flexion angle	0	30	60	90	120
Mean implant gap in FA (mm)	1.66 ± 1.48 (−1.2 to 5.7)	2.67 ± 1.96 (−0.6 to 6.7)	1.68 ± 2.15 (−1.0 to 7.8)	1.06 ± 1.87 (−2.0 to 7.1)	0.75 ± 1.73 (−0.9 to 6.9)
Mean implant gap in AA (mm)	2.38 ± 1.84 (−0.4 to 5.7)	3.63 ± 2.45 (−0.9 to 7.8)	2.99 ± 2.58 (−2.3 to 7.2)	2.55 ± 2.11 (−0.5 to 7.7)	2.38 ± 2.21 (−1.3 to 8.6)
*p*	0.001	0.001	<0.001	<0.001	<0.001
CI	[0.29–1.17]	[0.39–1.56]	[0.69–1.94]	[0.96–2.02]	[1.10–2.15]

Abbreviations: AA, anatomic alignment; FA, functional alignment.

## DISCUSSION

The most important finding of this study was that soft tissue balance after implantation was achieved in FA TKA patients at all knee angles. In particular, the balance in the medial compartment, which is considered the most important, was close to the target value at all angles. This means that the gap values that were simulated and adjusted before osteotomy via the FA technique were accurately reproduced after implant placement. In clinical practice, creating an ideal and stable gap in every surgery is technically difficult to achieve using conventional methods. It is difficult to remove the osteophytes of the posterior femoral condyle prior to osteotomy, and the amount of osteotomy must be determined while anticipating how much the extension gap will increase when the posterior condyle is resected after some osteotomy is performed [10]. It is also difficult to resect the entire meniscus before osteotomy, and the effect of resection must also be considered. The amount and angle of osteotomy must be determined, taking into account any existing flexion contracture or hyperextension [22]. Often, the gap is so narrow that an additional osteotomy is needed, or the gap is so large that a thicker insert is required. In this study, the FA technique using Mako allowed us to confirm the accuracy of the technique by maintaining the simulated preoperative target gap even during the implant trial phase. Many reports suggest that the balance of FA improves compared with that of MA and KA, which is consistent with the results of this study [5, 26]. However, these were simulation studies conducted before the osteotomy was performed, and they only evaluated extension and flexion positions. If an appropriate implant gap can be obtained with the use of FA as in the case of this study, there is no need for additional soft tissue release or osteotomies [15]. As a result, postoperative patient‐reported outcomes are expected to improve [4, 15].

Mid‐flexion instability has been reported as a cause of instability in stair climbing and other postural positions [16, 25, 27]. This is one of the failures of TKA, and maintaining stability at all angles is one of its challenges. Clark et al. [5] reported that good balance can be achieved in extension and flexion in pre‐osteotomy simulations; however, there have been no reports on balance in mid‐flexion after FA thus far. In the present study, gap values, especially in the medial compartment, did not open up in the mid‐flexion position, and mid‐flexion instability was not observed. This could be due to several factors. First, the subject of this study was a CR‐type. If the PCL is retained, there is less mid‐flexion instability than in the resected PS‐type [8]. If the PCL is resected, it might be more open. Second, the current study used a plan based on a restricted KA, not an MA. The femoral side was osteotomized valgus, and the tibial side was osteotomized varus, which tilted the articular plane medially. The elevation of the joint line on the medial side is the main cause of mid‐flexion instability [17]. The lowering of the joint line on the medial aspect may have allowed the MCL to function in the mid‐flexion position, resulting in a stable knee.

Comparing the balance between FA and AA, the gap was more open with flexion in AA than in FA in all medial, central, and lateral compartments; in AA, the extension gap was created, and then the tensioner was used to evaluate the extension and flexion gaps. The difference in the values determined the amount of osteotomy of the posterior femoral condyle so that the flexion gap was also equal. As a result, the implant gap was actually open in the flexion position. In contrast, in FA, the preoperatively evaluated flexion gap value was maintained at the implant gap. It is difficult to evaluate with a tensioner in a completely un‐osteotomized state. It can be influenced by the residual posterior condyle; although a relatively good balance was obtained with AA, the FA simulation was more accurate.

In the lateral compartment, the gap for AA was greater than that for FA. This shows that AA was looser than the FA on the lateral side. The most likely cause of the greater gap in AA is the effect of the osteotomy angle of the tibia. In terms of patient background, both FA and AA patients had greater varus with an MPTA of 84°. AA patients underwent osteotomies with 2° of varus, and FA patients underwent osteotomies with 3.5° of varus. In AA, the lateral side osteotomy is greater than that in FA, resulting in lateral laxity. The lateral femoral side is less affected than the tibial side, since the varus‐valgus deformity on the femoral side is smaller than that on the tibial side. The average LDFA for the patients in this study was between 88.5° and 88.6°. This is likely why lateral laxity is created in AA relative to FA. However, the lateral gap in flexion was originally reported to be open in healthy subjects [5, 12]. In addition, it has been reported that a greater lateral gap in flexion preserves a better range of motion and normal kinematics. However, if the lateral gap is too wide, impingement of the post may occur in the PS implant. The ideal gap should be a subject for further discussion.

Although FA and AA were compared in this study, these results can be used to predict the results of MA. In MA, the joint line may be elevated on the medial side; therefore, there is a possibility that the mid‐flexion gap will widen even more than that in the AA group. In addition, because the osteotomy of the tibia involves a large amount of lateral cuts, there is a possibility that the gap on the lateral side will be larger than that in the AA group.

The strength of this study is that it evaluated the component gap after implantation at multiple points of the knee joint's range of motion. To date, only reports on the extension gap and flexion gap have been published. In addition, it is a direct comparison of the systematic approach AA and personalized approach FA using the same protocol. Based on the data on soft tissue balance during surgery, FA is superior to AA. However, this study has limitations. First, this was a sequential cohort study, and patient allocation was based on the timing of surgery. Second, this case study was limited to varus knees. It is unclear how the results of this study will apply to valgus knees. Thirdly, the data on knee phenotypes were limited to MPTA and LDFA in this study. If CT data were available for all cases, it would have been possible to evaluate the phenotypes in greater detail as reported by Hirschmann et al. and to clarify potential differences in bone morphology and gender [3, 11]. Fourthly, the two groups used different tools, robots, and navigation systems. Lastly, this study did not evaluate functional outcomes, such as gait stability or pain reduction.

## CONCLUSION

Compared with AA, FA achieved balance at all knee flexion angles. FA can achieve a well‐balanced knee with high reproducibility and may allow the surgeon to consistently obtain better balance.

## AUTHOR CONTRIBUTIONS

Conceptualization: Hironari Masuda and Takumi Nakagawa. Data analysis and interpretation: Noriaki Arai, Yutoshi Osaki and Hironari Masuda. Statistical analysis: Shuji Ando. Writing—original draft: ST. All authors critically reviewed and revised the manuscript draft and approved the final version for submission.

## CONFLICT OF INTEREST STATEMENT

Authors Seikai Toyooka and Takumi Nakagawa have received speaker and consultant honoraria from Stryker. The remaining authors declare no conflicts of interest.

## ETHICS STATEMENT

This study was performed in line with the principles of the Declaration of Helsinki. Approval was granted by the Ethics Committee of Teikyo University (approval number: 19‐174‐2). Patients signed informed consent regarding publishing their data and photographs.

## Data Availability

The authors will make data and materials supporting the results or analyses presented in the paper available upon reasonable request.

## References

[1] Arai N , Toyooka S , Masuda H , Kawano H , Nakagawa T . Kinematic alignment achieves a more balanced total knee arthroplasty than mechanical alignment among CPAK type I patients: a simulation study. J Clin Med. 2024;13:3596. 38930125 10.3390/jcm13123596 PMC11204712

[2] Chang JS , Kayani B , Wallace C , Haddad FS . Functional alignment achieves soft‐tissue balance in total knee arthroplasty as measured with quantitative sensor‐guided technology. Bone Joint J. 2021;103‐b:507–514. 10.1302/0301-620X.103B.BJJ-2020-0940.R1 33467917

[3] Chelli S , Rudyy T , Avram GM , Huegli RW , Amsler F , Hirschmann MT . Gender‐based differences exist in the functional knee phenotypes classification of the osteoarthritic knee. Knee Surg Sports Traumatol Arthrosc. 2024;32:2505–2515. 38415864 10.1002/ksa.12082

[4] Choi BS , Kim SE , Yang M , Ro DH , Han HS . Functional alignment with robotic‑arm assisted total knee arthroplasty demonstrated better patient‐reported outcomes than mechanical alignment with manual total knee arthroplasty. Knee Surg Sports Traumatol Arthrosc. 2023;31:1072–1080. 36378291 10.1007/s00167-022-07227-5

[5] Clark G , Steer R , Wood D . Functional alignment achieves a more balanced total knee arthroplasty than either mechanical alignment or kinematic alignment prior to soft tissue releases. Knee Surg Sports Traumatol Arthrosc. 2023;31:1420–1426. 36116071 10.1007/s00167-022-07156-3 PMC10050049

[6] Deep K , Picard F , Clarke JV . Dynamic knee alignment and collateral knee laxity and its variations in normal humans. Front Surg. 2015;2:62. 26636090 10.3389/fsurg.2015.00062 PMC4658436

[7] Erard J , Olivier F , Kafelov M , Servien E , Lustig S , Batailler C . Enhancing Soft Tissue Balance: Evaluating Robotic‐assisted Functional Positioning in Varus Knees Across Flexion and Extension With Quantitative Sensor‐guided Technology. Knee Surgery, Sports Traumatology, Arthroscopy. 2024;32:2318–2327. 10.1002/ksa.12255 38738818

[8] Hino K , Kutsuna T , Oonishi Y , Watamori K , Kiyomatsu H , Iseki Y , et al. Assessment of the midflexion rotational laxity in posterior‐stabilized total knee arthroplasty. Knee Surg Sports Traumatol Arthrosc. 2017;25:3495–3500. 27246993 10.1007/s00167-016-4175-1

[9] Hirschmann MT , Becker R , Tandogan R , Vendittoli PA , Howell S . Alignment in TKA: what has been clear is not anymore! Knee Surg Sports Traumatol Arthrosc. 2019;27:2037–2039. 31190246 10.1007/s00167-019-05558-4

[10] Holst DC , Doan GW , Angerame MR , Roche MW , Clary CW , Dennis DA . What is the effect of posterior osteophytes on flexion and extension gaps in total knee arthroplasty? A cadaveric study. Arthroplast Today. 2021;11:127–133. 34522740 10.1016/j.artd.2021.08.007 PMC8427272

[11] Howell SM , Kuznik K , Hull ML , Siston RA . Results of an initial experience with custom‐fit positioning total knee arthroplasty in a series of 48 patients. Orthopedics. 2008;31:857–863. 18814593 10.3928/01477447-20080901-15

[12] Itou J , Itoh M , Kuwashima U , Okazaki K . Lateral joint tightness in flexion following cementless mobile‐bearing total knee arthroplasty decreases patient‐reported outcome measures and postoperative range of motion. J ISAKOS. 2023;8:332–337. 37321294 10.1016/j.jisako.2023.06.003

[13] Jacobs CA , Christensen CP . Factors influencing patient satisfaction two to five years after primary total knee arthroplasty. J Arthroplasty. 2014;29:1189–1191. 24534535 10.1016/j.arth.2014.01.008

[14] Kahlenberg CA , Nwachukwu BU , McLawhorn AS , Cross MB , Cornell CN , Padgett DE . Patient satisfaction after total knee replacement: a systematic review. HSS J. 2018;14:192–201. 29983663 10.1007/s11420-018-9614-8 PMC6031540

[15] Lee JH , Kwon SC , Hwang JH , Lee JK , Kim JI . Functional alignment maximises advantages of robotic arm‐assisted total knee arthroplasty with better patient‐reported outcomes compared to mechanical alignment. Knee Surg Sports Traumatol Arthrosc. 2024;32:896–906. 38454836 10.1002/ksa.12120

[16] Longo UG , Candela V , Pirato F , Hirschmann MT , Becker R , Denaro V . Midflexion instability in total knee arthroplasty: a systematic review. Knee Surg Sports Traumatol Arthrosc. 2021;29:370–380. 32133537 10.1007/s00167-020-05909-6

[17] Luyckx T , Vandenneucker H , Ing LS , Vereecke E , Ing AV , Victor J . Raising the joint line in TKA is associated with mid‐flexion laxity: a study in cadaver knees. Clin Orthop Relat Res. 2018;476:601–611. 29443845 10.1007/s11999.0000000000000067 PMC6260050

[18] MacDessi SJ , Griffiths‐Jones W , Harris IA , Bellemans J , Chen DB . Coronal Plane Alignment of the Knee (CPAK) classification: a new system for describing knee phenotypes. Bone Joint J. 2021;103–b:329–337. 10.1302/0301-620X.103B2.BJJ-2020-1050.R1 PMC7954147 33517740

[19] MacDessi SJ , Oussedik S , Abdel MP , Victor J , Pagnano MW , Haddad FS . The language of knee alignment: updated definitions and considerations for reporting outcomes in total knee arthroplasty. Bone Joint J. 2023;105‐b:102–108. 10.1302/0301-620X.105B2.BJJ-2022-1345 36722056

[20] Okada S , Yagi M , Taniguchi M , Motomura Y , Okada S , Fukumoto Y , et al. Investigation of the relationship between soft tissue stiffness and maximum knee extension angle in patients with knee osteoarthritis. J Biomech. 2025;182:112582. 39938442 10.1016/j.jbiomech.2025.112582

[21] Oussedik S , Abdel MP , Victor J , Pagnano MW , Haddad FS . Alignment in total knee arthroplasty. Bone Joint J. 2020;102‐b:276–279. 10.1302/0301-620X.102B3.BJJ-2019-1729 32114811

[22] Sappey‐Marinier E , Fernandez A , Shatrov J , Batailler C , Servien E , Huten D , et al. Management of fixed flexion contracture in primary total knee arthroplasty: recent systematic review. SICOT‐J. 2024;10:11. 38530205 10.1051/sicotj/2024007 PMC10964851

[23] Seito N , Suzuki K , Mikami S , Uchida J , Hara N . The medial gap is a reliable indicator for intraoperative soft tissue balancing in posterior‐stabilized total knee arthroplasty. Knee. 2021;29:68–77. 33578283 10.1016/j.knee.2021.01.013

[24] Toyooka S , Osaki Y , Masuda H , Arai N , Miyamoto W , Ando S , et al. Distribution of coronal plane alignment of the knee classification in patients with knee osteoarthritis in Japan. J Knee Surg. 2023;36:738–743. 35114721 10.1055/s-0042-1742645

[25] Vajapey SP , Pettit RJ , Li M , Chen AF , Spitzer AI , Glassman AH . Risk factors for mid‐flexion instability after total knee arthroplasty: a systematic review. J Arthroplasty. 2020;35:3046–3054. 32532482 10.1016/j.arth.2020.05.026

[26] Van de Graaf VA , Chen DB , Allom RJ , Wood JA , MacDessi SJ . Functional alignment in total knee arthroplasty best achieves balanced gaps and minimal bone resections: an analysis comparing mechanical, kinematic and functional alignment strategies. Knee Surg Sports Traumatol Arthrosc. 2023;31:5118–5127. 37789215 10.1007/s00167-023-07567-w

[27] Vince K . Mid‐flexion instability after total knee arthroplasty: woolly thinking or a real concern? The bone & joint. Journal. 2016;98:84–88. 10.1302/0301-620X.98B1.36445 26733649

[28] Winnock de Grave P , Luyckx T , Claeys K , Tampere T , Kellens J , Müller J , et al. Higher satisfaction after total knee arthroplasty using restricted inverse kinematic alignment compared to adjusted mechanical alignment. Knee Surg Sports Traumatol Arthrosc. 2022;30:488–499. 32737528 10.1007/s00167-020-06165-4 PMC8866329

